# Difficult Extractions

**Published:** 1906-10

**Authors:** L. G. Noel

**Affiliations:** Nashville, Tenn.


### DIFFICULT EXTRACTIONS.

By L. G. Noel, D. D. S., Nashville, Tenn.

We frequently hear the statement made by our professional friends and made with manifest pride, "I seldom ever extract teeth;" and "I haven't extracted half a dozen teeth in as many years." It is a commendable pride that thus vaunts itself upon salvation rather than destruction, and a most successful and remunerative practice may be conducted by a dentist who never extracts teeth; but if he discharges his duty toward his patients, he mus often send them to some one else to have this operation performed, as the duties of the dental profession often call for it most imperatively.

One of the errors most frequently made is hastening to apply the forceps as soon as the tooth, or root, has been condemned to extraction, without making sufficiently careful examination of the case. The fracture of teeth, or roots, often results from the too hasty and inconsiderate application of force—a mishap that may be avoided by first making a thorough study of the case. Frequently too much force is applied, or too much cutting of the alveolar process, when a testing of the solidity of the root, before applying the forceps, would have revealed its weakness and perhaps have led to a different method altogether—possibly the elevator instead of the forceps. The careless operator has often felt shame for the heroic bite he has taken with the alveolar forceps to extract a contemptible little stump that he could easily have overturned with the elevator.

The exploring needle in the hands of a skillful dental surgeon, whose touch has been "educated," locates hidden roots, abscess cavities, necrotic tissues, and phagedenic destruction of bone. Its revelations are swifter and surer, under some conditions, than the X-ray diagnosis.

In locating malplaced and unerupted wisdom teeth this method is unequaled. But there are comparatively few dental surgeons provided with the facilities or equipped with the skill and training in the art of skyeograph pictures necessary for making this kind of diagnosis. No one, we think, will dispute the value of this insight into the hidden things of nature; and when all other means have failed to give the operator a fairly clear mental picture of the case, before proceeding we should resort to this, even if it should be necessary to call in the aid of a specialist in the work.

Your essayist has had some experience in extracting impacted wisdom teeth, and he knows that in some cases it is a surgical operation requiring a high order of skill as well as one that taxes the patience, ingenuity, and ability of the dental surgeon. In many instances the lower third molar lies tilted anteriorly, with its masticating surface in opposition with the distal surface of the second molar. We find these cases presenting all angles of inclination. Sometimes the masticating surface is in close adaptation to the distal surface of the second molar, and the third molar lies with its longitudinal axis in a horizontal relation to the other teeth. When this is the case, the extraction of the tooth is almost impossible without first cutting away the bone overlying the roots and down beside the tooth, lingually and buccally, enough to admit of grasping it with the forceps.

Efforts to remove these teeth without such preliminary measures are sure to result in failure and often in fracturing the bone and tooth, causing the fracture of the crown of the adjoining molar, and resulting, after all, in disaster and failure.

In these cases I prefer to any other general anæsthetic ether; for nitrous oxide is too evanescent in its effects, and produces a spasmodic contraction of the temporal and masseter muscles that is very undesirable. With many patients I find the injection of nirvanin all that is necessary. When this local anæsthetic is employed, the syringe should be in good order, having all its fittings tight, so that the fluid can be injected into the peridental membrane slowly until the gum is whitened by the pressure of fluid in the tissues. The needle should be inserted at several points about the field of operation, and time should be allowed for the dissemination of the drug through the tissues. I usually wait from ten to fifteen minutes after injecting the fluid before commencing my operation.

For cutting away the bone I use bud-shaped burs. These may be for both the direct and the right-angle hand piece. The hemorrhage following the cutting of the gum and periosteum is quickly checked by applying adrenalin chloride. The progress of the work of cutting away the bone should be noted from time to time by syringing and wiping out the wound with absorbent cotton. When the bone has been removed from over the distal root, and sufficient of the crown has been exposed for obtaining a hold, it may be grasped with a pair of Hutchinson's forceps and removed. In some cases I have worked the cow horn forceps to advantage, they being a powerful lever.

The wound will require daily syringing with dioxygen until granulations have filled the cavity, and even then the patient should be instructed to use constantly an antiseptic mouth wash.

Another class of cases that may be extracted piecemeal, by the use of the bud and flame-shaped burs in the dental engine, are the so-called "goose quill roots," where caries has followed down the pulp chamber and destroyed the dentine until little but a shell of cementum remains. This shell may be burred out with the flame-shaped cavity burs; and when a portion of one side has been removed the remainder may be dislodged with a small sickle scaler, a hoe-shaped excavator, or almost any hooked instrument that will enter the cavity. This is better surgery than biting through the alveolar process and cutting a deep trench in the bone with a pair of alveolar forceps.

Many of these roots that have abscessed will be found so loosened by disease as to be easy to dislodge with a dental elevator.

I shall not feel that I have discharged my duty if I do not, before closing, inveigh against the practice of using so recklessly the alveolar cutting forceps for biting out these roots. We all know from observation that in so doing we remove more bone than would be taken away by the natural process of reabsorption; for we have all been annoyed by these deep trenches that we find so difficult to restore with any artificial substitute, and we have frequently had occasion to regret the sunken and pinched expression thus imparted to the face. When it is our purpose to restore the lost teeth with bridge work, especially in the front of the mouth, we should avoid this reckless destruction of bone.

